# Diet- and Sleep-Based Approach for Cardiovascular Risk/Diseases

**DOI:** 10.3390/nu15173668

**Published:** 2023-08-22

**Authors:** Masahiko Kato

**Affiliations:** Division of School of Health Science, Department of Pathobiological Science and Technology, Faculty of Medicine, Tottori University, Yonago 683-8503, Japan; mkato@tottori-u.ac.jp

Central sleep apnea represented by Cheyne–Stokes Respiration (CSR) is frequently observed in heart failure (HF) patients, and its severity has been reported to be associated with morbidity and mortality in patients with HF [1]. CSR is caused by an increased sensitivity of the chemoreceptors; however, there is little evidence on whether controlling this respiration can improve the prognosis of HF patients. Additionally, HF patients are known to suffer from malnutrition due to chronic systemic inflammation [2], and decreased activities of daily living (ADL). While several useful indicators for evaluating the degree of nutritional disorders have been reported recently [3,4,5], there are no reports of randomized controlled trials (RCTs) proving the improvement of HF prognosis through nutritional intervention.

The study by Abulimiti et al. investigated the association between sleep disordered breathing reflecting the severity of HF and malnutrition causing frailty [6]. They divided acute decompensated HF (ADHF) patients into four groups based on the presence or absence of CSR and malnutrition and compared their long-term prognosis. The results showed that the group with CSR and malnutrition had the worst prognosis. As the tendency for malnutrition becomes stronger with increasing age in HF patients, they conducted multivariate analysis adjusting for age, creatinine, and brain natriuretic peptides (BNP). However, even with these adjustments, the group with CSR and malnutrition still showed a strong association with all-cause mortality. Although the average age in this study was 62 years old, it remains uncertain whether similar results would be obtained when targeting elderly ADHF patients (70–90 years old) from developed countries in the real world. Nonetheless, in ADHF patients in their 50s and 60s, the importance of CSR and malnutrition is presumed to outweigh age. Further studies targeting very elderly individuals will shed light on whether similar results are obtained, and the potential for improved prognosis through the control of CSR and nutritional interventions will be eagerly anticipated.
